# Modified Qing’ e Pills exerts anti-osteoporosis effects and prevents bone loss by enhancing type H blood vessel formation

**DOI:** 10.3389/fendo.2022.998971

**Published:** 2022-09-06

**Authors:** Junjie Lu, Desheng Hu, Chen Ma, Xiaojuan Xu, Lin Shen, Jianhui Rong, Jia Zhao, Bo Shuai

**Affiliations:** ^1^ Department of Integrated Traditional Chinese and Western Medicine, Union Hospital, Tongji Medical College, Huazhong University of Science and Technology, Wuhan, China; ^2^ School of Chinese Medicine, The University of Hong Kong, Pokfulam, Hong Kong, SAR China

**Keywords:** osteoporosis, bone loss, angiotensin, alternative medicine, type H blood vessels, vessel formation

## Abstract

**Objective:**

To explore whether the modified Qing’ e Pills (MQEP) exerts anti-osteoporotic effects and prevents bone loss by enhancing angiogenesis.

**Methods:**

Network pharmacology was used to assess whether MQEP has a pro-angiogenic capacity and to predict its potential targets. Human umbilical vein endothelial cells were treated with glucocorticoids and MQEP to assess cell viability. The expression of angiotensin II type 1 receptor, angiotensin II type 2 receptor, and angiotensin converting enzyme, which are associated with the activation of the renin-angiotensin-aldosterone system, and the expression of vascular endothelial growth factor and hypoxia-inducible factor 1 alpha, which are associated with the formation of type H blood vessels, were examined by western blot and RT-qPCR. Thereafter, the glucocorticoid-induced osteoporosis model was established and intervened with MQEP. Femur scanning was performed with micro-computed tomography; trabecular spacing, trabecular thickness, and trabecular number were observed and calculated; the expression of nuclear factor-kappa B ligand and osteoprotegerin was detected by ELISA, and the ratio was calculated to evaluate the degree of bone resorption. Finally, type H blood vessels that were highly coupled to osteogenic cells were identified by immunohistochemistry staining and flow cytometry.

**Results:**

This is the first study to reveal and confirm that MQEP could prevent bone loss in glucocorticoid-induced osteoporosis by promoting the expression of hypoxia-inducible factor 1 alpha and vascular endothelial growth factor, which are highly associated with type H blood vessel formation. *In vitro* experiments confirmed that MQEP could effectively promote the proliferation of vascular endothelial cells and alleviate glucocorticoids-induced activation of the renin-angiotensin-aldosterone system, thereby reducing vascular injury.

**Conclusion:**

MQEP exerts anti-osteoporosis effects and prevents bone loss by alleviating vascular injury caused by renin-angiotensin-aldosterone system activation and promoting type H blood vessel formation.

## Introduction

Osteoporosis is pathologically characterized by reduced bone mass, destruction of the bone microarchitecture, and decreased bone strength. Osteoporosis has no specific symptoms at the initial stage and is often ignored by patients, often only receiving attention after it has caused a fracture (1). In addition to postmenopausal women being prone to osteoporotic fractures, certain patients who require long-term glucocorticoid (GC) or immunosuppressive drugs are at significantly increased risk of osteoporotic fractures (2, 3). Patients with osteoporotic fractures are mostly senior citizens or those with an underlying poor physical condition. Once a fracture occurs, it increases the patient’s risk of disability and mortality. The main treatment options are currently divided into surgical and conservative treatments. Patients with osteoporotic fractures are usually above 65 years or have underlying disease, poor blood supply, and vascular conditions leading to delayed healing or non-healing, and refracture often occurs after intervention. Therefore, improving vascular conditions and promoting angiogenesis through various pathways have become effective strategies to prevent bone loss and alleviate osteoporosis.

Various factors influence microvascular-related bone loss conditions, the main ones being the regulation of the renin-angiotensin-aldosterone system (RAAS) (4) and the nuclear factor-kappa B (RANK) ligand (L)/RANK/osteoprotegerin (OPG) system which is closely related to bone metabolism (5). Activation of the RAAS leads to microvascular dysfunction and inhibits the microvascular formation. In addition, an upregulation of the RANKL/OPG ratio leads to a decrease in the number and viability of osteoblasts and induces osteoblast apoptosis, resulting in a negative balance between bone resorption and bone formation, imbalanced bone remodeling, and bone loss. Activation of the RANKL/RANK/OPG system causes greater bone resorption than bone formation, leading to increased bone loss and osteoporosis (6). In contrast, the activation of the RAAS leads to local inhibition of angiogenesis and microvascular dysfunction (7). The activation of these two signaling systems occurs synergistically, exacerbating the rate of disease progression and leading to malignant progression.

A high expression of endomucin (EMCN) and platelet endothelial cell adhesion molecule-1 (PECAM-1/CD31) was defined as type H blood vessels (8). Type H blood vessels have been shown to promote bone formation. Several factors, including hypoxia-inducible factor 1 alpha (HIF-1a) and vascular endothelial growth factor (VEGF), are involved in angiogenesis and osteogenic coupling (9). Type H blood vessels were reduced in ovariectomized mice with simulated postmenopausal osteoporosis. Also, they show an age-dependent decrease, with advanced age implying a lower abundance of these vessels. Hence, type H blood vessels is a marker of bone loss; by inducing an increase in type H blood vessels, bone mass can be effectively improved, bone loss can be significantly prevented, and fractures can swiftly heal (10).

The modified Qing’ e Pills (MQEP) is an herbal compound used in China for over a thousand years and as a complementary treatment for primary osteoporosis with good clinical efficacy (11). Some studies have shown that MQEP can improve bone microstructure and biomechanics in ovariectomized osteoporosis models, and this effect is achieved by increasing β-catenin protein expression (12). In addition, MQEP reportedly has estrogen-like effects with a high affinity for estrogen receptor α and can be used as a natural phytoestrogen (13). Finally, MQEP exerts beneficial effects in improving bone microstructure and slowing bone loss; however, its potential mechanism of action on blood vessels is unclear. In this study, we explored the novel mechanism by which MQEP acts on blood vessels to improve blood supply conditions, promote vascular proliferation, and thus achieve bone repair and reconstruction. This may provide an emerging theoretical basis for future treatment and more evidence to support the use of MQEP.

## Materials and methods

### Formula of traditional Chinese medicine decoctions

According to the original formula in the Dictionary of Chinese Medical Formulas, MQEP consists of *Eucommia ulmoides* (960 g), *Fructus psoralease* (480 g), *Semen juglandis* (300 g), and *Allium sativum* (240 g). All the herbs were washed and placed in a multifunctional extractor. Thereafter, the herbs were immersed in double distilled water (five times their volume) for 2 h, followed by boiling for 2 h. After filtering the boiled decoction, the herbal residues were boiled twice for 1 h each time, and the resulting boiled decoction was filtered, combined with the first filtrate, and condensed to a thick paste (100%), which was then added to 95% ethanol (three-fold of its volume) under stirring. After standing for 24 h, the solution was filtered to recover the ethanol fraction, and a 5 g/mL concentrate was obtained. The yield of MQEP extraction was 70%. The mice received a daily intragastric MQEP dose of 1.7 g/kg body weight diluted with distilled water. The MQEP dose calculation was performed in accordance with the guidelines correlating the dose equivalents between humans and laboratory animals based on the ratio of the body surface area (14).

### Experimental animals and tissue collection

Twenty-four C57BL/6J male mice were purchased from Beijing Charles River Experimental Animal Technology, the average age is 7 weeks and the weight is 22-25g. Both of them were raised in pathogen-free facilities under a 12-h light/dark cycle and provided with ample plastic bedding and clean water. Mice were randomly divided into three groups (n = 8 per group) as follows: control group (intraperitoneal injection of normal saline and intragastric administration of normal saline), model group (15, 16) (intraperitoneal injection of dexamethasone (DEX) (MedChemExpress, NJ, USA) and intragastric administration of normal saline), and MQEP group (intraperitoneal injection of DEX and intragastric administration of MQEP). The model group was established by intraperitoneal injection of 10 mg/kg DEX thrice a week for 3 months. The MQEP group received intragastric administration of MQEP and an intraperitoneal injection of DEX. Three months later, all mice were anesthetized with pentobarbital sodium and killed by dislocation of cervical vertebra. Femurs were collected, and the soft tissues were separated. A single cell suspension was prepared from the femurs for flow cytometry. The femurs were fixed in 4% paraformaldehyde solution and maintained in Dulbecco’s phosphate-buffered saline (DPBS) (pH 7.2) (Servicebio, Wuhan, China) until micro-computed tomography (CT) analysis, histomorphometry, and immunohistochemistry were performed.

### Micro-CT

The specimens were scanned using a Bruker Micro-CT Skyscan 1276 system (Kontich, Belgium). The scan settings were as follows: voxel size 6.533712 μm1, medium resolution, 70 kV,200 mA, 1 mm Al filter, and an integration time of 525 ms. Microstructure measurements were calibrated to the manufacturer’s calcium hydroxyapatite phantom. The analysis was performed using the manufacturer’s evaluation software. Reconstruction was accomplished using NRecon (version 1.7.4.2)2. 3 dimensional images were obtained from contoured 2 dimensional images using methods based on the distance transformation of the original grayscale images (CTvox; version 3.3.0); 3 dimensional and 2 dimensional analyses were performed using CT Analyzer software (Bruker, version 1.18.8.0).

### Elisa

Concentrations of RANKL and OPG in mice in femur was measured using a commercial mouse RANKL or OPG ELISA kit (Elabscience, Wuhan, China). the femurs were minced into small pieces and rinsed in ice-cold DPBS and weighed, then homogenized in PBS (1:9) with a homogenizer (Servicebio, Wuhan, China). The homogenates were then centrifuged for 10 min at 5000×g at 4°C to get the supernatant. Then follow these Assay procedures: 1) Determine wells for diluted standard, blank and sample. Add 25 μL each dilution of standard, blank and sample into the appropriate wells. Cover the plate with the sealer provided in the kit. Incubate for 90 min at 37°C; 2) Decant the liquid from each well, immediately add 50 μL of Biotinylated Detection Ab working solution to each well. Cover the plate with a new sealer. Incubate for 1 hour at 37°C; 3) Decant the solution from each well, add 350 μL of wash buffer to each well. Soak for 1 min and aspirate or decant the solution from each well and pat it dry against clean absorbent paper. Repeat this wash step 3 times; 4) Add 50 μL of HRP Conjugate working solution to each well. Cover the plate with a new sealer. Incubate for 30 min at 37°C; 5) Decant the solution from each well, repeat the wash process for 5 times; 6) Add 50 μL of Substrate Reagent to each well. Cover the plate with a new sealer. Incubate for about 15 min at 37°C. Protect the plate from light; 7) Add 25 μL of Stop Solution to each well; 8) Determine the optical density of each well at once with a micro-plate reader (Molecular Devices, San Jose, CA, USA) set to 450 nm.

### Immunohistochemistry staining

Three sets of fresh femurs were collected for immunofluorescence, and soft tissues were separated and immediately fixed overnight in ice-cold 4% paraformaldehyde. The femurs were decalcified using 0.5 M EDTA at 4 °C with continuous shaking. All samples were embedded in the OCT compound and cut into 25-μm-thick sagittal sections using a cryostat (Leica Biosystems, Germany). Next, sections were treated with 0.2% Triton X-100 for 10 min, then closed with 5% serum for 30 min at room temperature, and incubated overnight at 4°C with the following antibodies: CD31 (1:100; Servicebio, Wuhan, China) and EMCN (Santa Cruz Biotechnology, USA). EMCN self-coupled FITC fluorescence and CD31 were visualized using Cy3-coupled secondary antibodies (1:100; ProteinTech, Wuhan, China) against the corresponding species. The nuclei were stained with DAPI (Servicebio, Wuhan, China). All samples were observed under a fluorescence microscope (Olympus-life science, Japan), and the mean fluorescence intensity was quantified using Image J software.

### Flow cytometry

After removal of the surrounding connective tissue, femurs were dissected from the three groups of mice. Femurs were clipped in DPBS digested with 2.5 mg/mL collagenase A (Biosharp, Hefei, China) and enzymatically with 1 unit/mL dispersible enzyme I (Biosharp, Hefei, China) and kept under gentle agitation at 37°C for 15 min. The obtained cell suspension was filtered using a 40-um cell strainer (Biosharp, Hefei, China) and washed with DPBS. After washing, the cells were incubated with FITC-conjugated EMCN antibody (Santa Cruz Biotechnology, USA) and APC-conjugated CD31 antibody (BioLegend, San Diego, CA) for 30 min at room temperature. Thereafter, the cells were resuspended in DPBS. All samples were analyzed using the CytoFLEX LX Flow Cytometry System (CytoFLEX, Backman Counter, CA, USA) and CytExpert 2.4 Software.

### Cell culture and treatment

Human umbilical vein endothelial cells (HUVEC) were obtained from the laboratory of the Department of Integrated Traditional Chinese and Western Medicine, Union Hospital, Tongji Medical College, Huazhong University of Science and Technology. Cells were cultured in Dulbecco’s modified Eagle medium (Gibco, Grand Island, NY, USA) supplemented with 10% fetal bovine serum (Gibco) at 37°C in a 5% CO_2_ humidified cell incubator. Three groups of cells were treated as follows: the control group (no processing), model group (pre-processing with 100 μM DEX), and MQEP group (pre-processing with DEX and intervened with 0.5 mg/ml.).

### Cell viability and toxicity assay

The Cell Counting Kit-8 (GlpBio Technology, CA, USA) was used to determine cell viability. HUVEC cells were seeded in 96-well plates at a density of 3×10^3^ cells/well and incubated with DEX and MQEP for indicated time periods at 37°C. After treatment, 10 μL cell counting kit-8 solution was added to the culture and incubated at 37°C for 1 h. Absorbance was measured at 450 nm using a microplate reader (Molecular Devices, San Jose, CA, USA).

### Western blot analysis

HUVEC (1×10^6 cells/well) were seeded in 6-well plates for 48 h and treated with DEX and MQEP for 48 h. Cells were washed twice with DPBS and lysed with RIPA lysis buffer (Servicebio, Wuhan, China) and phenylmethanesulfonyl fluoride (Servicebio, Wuhan, China). Protein loading buffer was added, and protein denaturation was performed at 95 °C for 15 min. Protein samples were separated by 10% or 12% 12% alkyl sulfate-polyacrylamide gel electrophoresis and transferred onto a polyvinylidene fluoride membrane (Merck Millipore, Darmstadt, Germany), which was blocked with 5% milk in Tris Buffer Solution with Tween 20 for 1 h and incubated with the primary antibody (β-actin, Servicebio, Wuhan, China; angiotensin II type 1 receptor (AT1R)/angiotensin II type 2 receptor (AT2R)/angiotensin converting enzyme (ACE), ABclonal, Wuhan, China; HIF-1a/VEGF, ProteinTech, Wuhan, China) at 4°C overnight. The membranes were washed with TBST three times and then incubated with HRP-conjugated secondary antibody at room temperature for 1.5 h. After washing with TBST three times, the membranes were incubated with ECL solution (Biosharp, Hefei, China), and images were obtained using a Gel Imaging System (Bio-Rad ChemiDoc™ XRS+). Protein levels were quantified using the ImageJ software.

### mRNA expression analysis

HUVEC (1×10^6 cells/well) were seeded in 6-well plates for 48 h and treated with DEX and MQEP for 48 h. After the cells were harvested, total RNA was extracted using TRIzol (Takara Biomedical Technology Co., Ltd., Japan) and trichloromethane (Sinopharm Chemical Reagent Co., Ltd., Shanghai). Next, according to the instructions of the SynScript III cDNA Synthesis Mix kit (Tsingke Biotechnology, Beijing, China), total RNA was reverse transcribed into cDNA and amplified using a fluorescence quantitative PCR instrument (Molarray A6000, Suzhou, China). Primer related information was displayed in **Table 1**. Amplification reactions were performed according to the manufacturer’s protocol (Vazyme, 2 × Acetaq Master Mix dye plus; Nanjing, China) under the reaction conditions used for amplification. The calculation was based on the 2 − ΔΔ CT method, and GAPDH was used as a reference to normalize expression and calculate the relative expression of each group. The following is the RNA sequence (**Table 1**).

**Table 1:** The RNA sequences of RT-qPCR.

Gene	Species	Gene ID	Sequence (5'-3')	UniProt ID
**AT1R**	Human	185	F : CAGCGTCAGTTTCAACCTGTACG	P30556
			R : GCAGGTGACTTTGGCTACAAGC	
**AT2R**	Human	186	F : GGGGGAGGTTGGACTGTAAT	P50052
			R : AGGGCACATTTGCACATACA	
**ACE**	Human	1636	F : CATCACCACAGAGACCAGCAAG	P12821
			R : CCGCTTGATAGTGGTGTTCTGC	
**HIF-1a**	Human	3091	F : TATGAGCCAGAAGAACTTTTAGGC	Q16665
			R : CACCTCTTTTGGCAAGCATCCTG	
**VEGF**	Human	7422	F : TTGCCTTGCTGCTCTACCTCCA	P15692
			R : GATGGCAGTAGCTGCGCTGATA	
**GAPDH**	Huamn	2597	F : GTCTCCTCTGACTTCAACAGCG	P04406
			R : ACCACCCTGTTGCTGTAGCCAA	

### Network pharmacology

The gene card, Online Mendelian Inheritance in Man, Pharmacogenetics and Pharmacogenomics Knowledge Base, and Therapeutic Target Database were searched for disease-associated target genes, and Venn diagrams were drawn using the R package (VennDiagram). TCMSP and ETCM databases were used to search for the main components of MQEP, and the top 10 compounds were analyzed for interactions with their target proteins. The STRING database and the software Cytospace were used to construct protein interaction networks, and the R 3.6.0 software was used to convert gene names to gene IDs which were converted into colorspace, stringi, DOSE, clusterProfiler, pathview, and ggplot2 assembly packages. Bubble maps and key signaling pathway maps were also obtained. The structures of *Isopsoralen* key chemical components were obtained from the PubChem database and optimized in 3D using ChemDraw software. The structures of the target proteins were downloaded from the PDB database (http://www.rcsb.org/) and routinely pre-processed using PyMOL software. Subsequently, molecular docking was performed using the AutoDock software with default values for all docking parameters.

### Statistical analysis

All data were expressed as the mean ± standard deviation. Statistical analyses were performed using the GraphPad Prism software (GraphPad Prism version 6.0), and Student’s t-test was used to compare the means of the two groups. Comparisons among multiple groups were performed using one-way ANOVA with Tukey’s test. Statistical significance was set at P < 0.05.

## Results

### Network pharmacology searches for signaling pathways and targets on which MQEP exerts anti-osteoporosis and bone loss prevention effects

**Figure 1:**
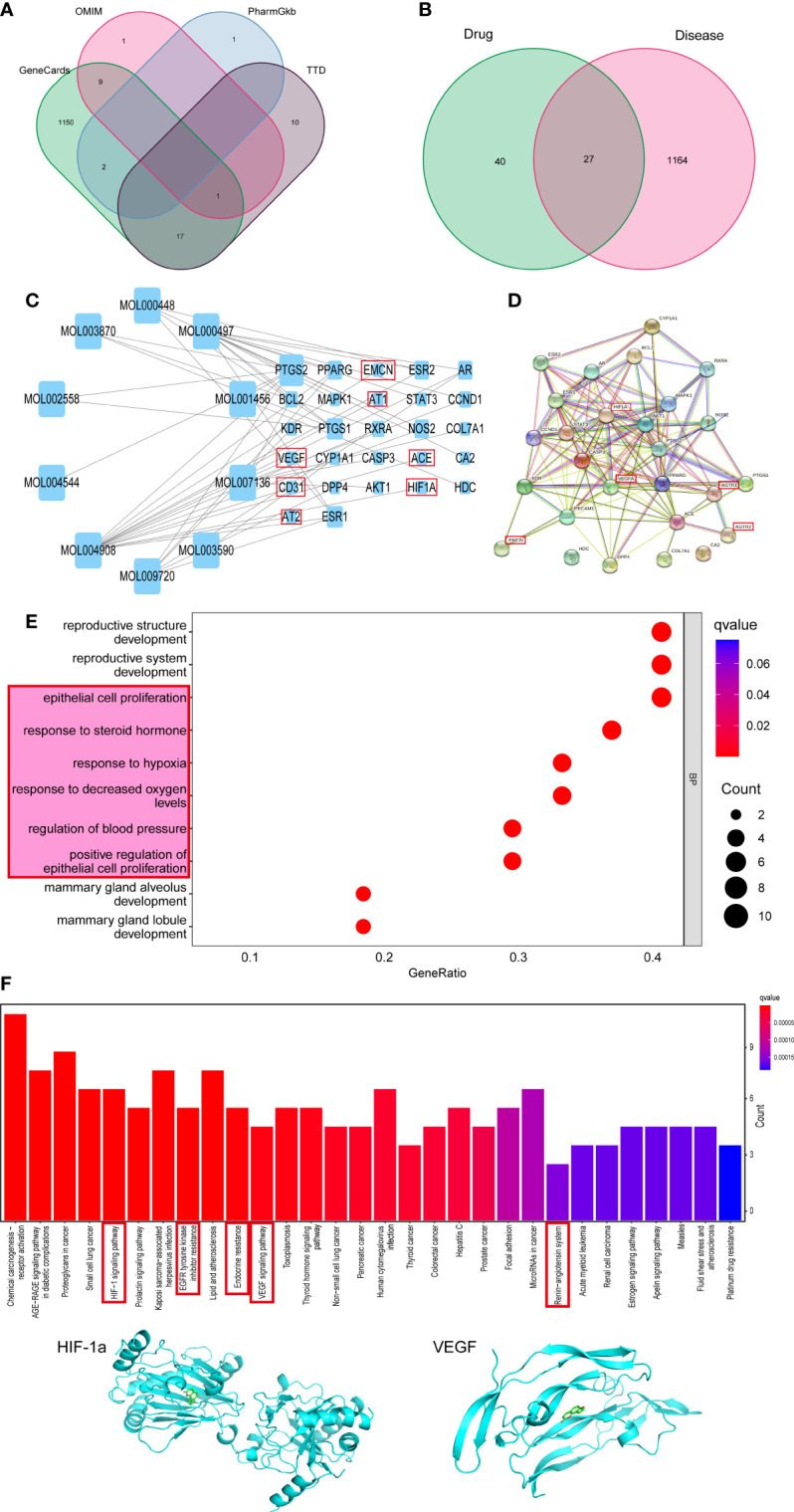
Predicting the targets MQEP by network pharmacology. **(A)** Targets for osteoporosis were retrieved from four disease databases. **(B)** An intersection of the MQEP and osteoporosis targets is the target on which MQEP exerts anti-osteoporosis and bone loss prevention effects. **(C)** The main components of MQEP were searched, and the top 10 compounds were analyzed for interactions with their target proteins of action using the TCMSP and ETCM databases. **(D)** The String database and Cytospace software were used to construct protein interaction networks. **(E, F)** The target on which MQEP exerts the anti-osteoporosis effect was obtained, and GO enrichment and KEGG enrichment were performed to obtain the main biological processes and signaling pathways. **(G)** Molecular docking was used to verify the target proteins with high affinity to *Isopsoralen*, the main component in MQEP.

To evaluate whether MQEP has the potential to prevent bone loss and exert anti-osteoporosis effects by promoting angiogenesis, we searched the Gene cards (https://www.genecards.org/), OMIM (https://omim.org/), PharmGkb (https://www.pharmgkb.org/), and TTD (http://bidd.nus.edu.sg/group/ttd/ttd.asp) databases for disease-related target genes using bone loss and osteoporosis as keywords, and obtained a total of 1,191 target genes related to osteoporosis and bone loss **(** **Figure 1A** **)**. In the previous study (17), we performed related work to quantify the aqueous decoction extract of MQEP by plant metabolomics technology and obtained all the small-molecule compound components. We identified the target genes of MQEP that exert anti-osteoporosis effects and prevent bone loss. After that, we searched the TCMSP (https://old.tcmsp-e.com/tcmsp.php) and ETCM databases (http://www.tcmip.cn/ETCM/) for the main components of MQEP, and the top 10 compounds were analyzed for interactions with their target proteins. A total of 27 target proteins were obtained **(** **Figure 1B** **)**, including CD31, EMCN, VEGF, AT1, AT2, ACE, and HIF-1a, all of which are involved in the pathological process of osteoporosis and bone loss and are the target proteins on which MQEP produces a marked effect. Next, we constructed a diverse protein interaction network of 27 target proteins **(** **Figure 1C** **)**. We observed that the more interactions between proteins, the denser the network, and the more critical the target protein’s role in the anti-osteoporosis and bone loss process. Among them, HIF-1a and VEGFA may be the key target proteins for which MQEP exerts its anti-osteoporosis and bone loss effects **(** **Figure 1D** **)**. We then used GO and KEGG enrichments to discover the specific biological functions of MQEP’s role in anti-osteoporosis and bone loss through GO enrichment and the pathways highly associated with anti-osteoporosis and bone loss through KEGG enrichment (**Supplement Figures 1A, B** **)**. The results showed that MQEP is involved in several biological processes such as endothelial cell proliferation, steroid hormone response, and blood pressure regulation, and regulates several signaling pathways such as AGE-RAGE, HIF-1a, VEGF, apelin signaling pathway, and renin-angiotensin system, all of which are highly related to angiogenesis **(** **Figures 1E, F** **)**. These signaling pathways may be responsible for the role of MQEP in promoting angiogenesis and may be the key pathways through which MQEP exerts its anti-osteoporosis and bone-loss effects. The previous quantitative analysis of the aqueous decoction extract of MQEP revealed that *Isopsoralen* is the main component of MQEP, HIF-1a and VEGF may be the key protein on which MQEP exerts its anti-osteoporosis and bone loss effects by promoting angiogenesis. Hence, we molecularly docked *Isopsoralen*, the main component of MQEP, with the main target proteins, HIF-1a and VEGF. There are 20 binding modes between *Isopsoralen* with HIF-1a, the affinity is between -8.1~-4.8, and the binding energy range is 0~32.714. There are also 20 binding modes between *Isopsoralen* with VEGF, the affinity is between -5.9~-4.3, and the binding energy range is 0~20. 267. And the best mode of action results was obtained **(** **Figure 1G** **)**. The results confirmed that *Isopsoralen*, the main component of MQEP, has a high affinity with the target proteins HIF-1a and VEGF.

### MQEP promoted HUVEC viability through multiple pathways

**Figure 2:**
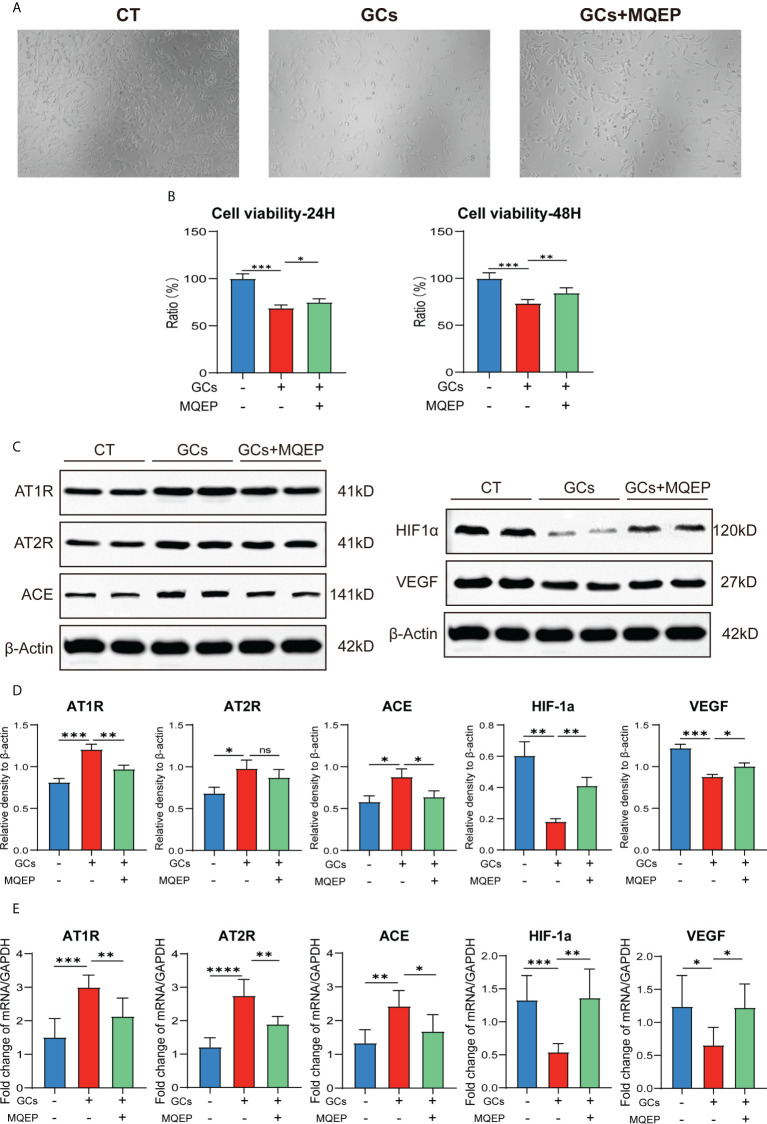
The intervention of MQEP reversed GC-induced activation of the RAAS and the downregulation of HIF-1a and VEGF expression. **(A)** The morphology of HUVEC cells was observed under the microscope after GC treatment and MQEP intervention. **(B)** CCK-8 assay was performed, the absorbance was measured at 450 nm, and cell viability was calculated. **(C, D)** The expression of several key factors of the RAAS and type H blood vessels were detected using Western blotting after treatment of HUVEC cells with MQEP for 48h. Images were quantified and statistically analyzed. **(E)** The expression of several key factors of the RAAS and type H blood vessels were detected using RT-qPCR after treating HUVEC cells with MQEP for 48h. ns, not significant, *p < 0.05, **p < 0.01, ***p < 0.001.

After GC treatment, the morphology of HUVEC changed from an irregular shuttle shape to an oval or round shape, and cell viability decreased. These changes occurred after MQEP intervention, with some cell morphology and viability restoration **(** **Figure 2A** **)**. We examined the expression of AT1R, AT2R, ACE, HIF-1a, and VEGF, which are highly correlated with the RAAS and type H blood vessel formation by western blot and RT-qPCR. We observed that AT1R, AT2R, and ACE, correlated with the RAAS system, were upregulated, while HIF-1a and VEGF, correlated with type H blood vessels, were downregulated after GC treatment **(** **Figures 2B–E** **)**. These results showed that the RAAS was activated, and type H blood vessel formation was inhibited after GC use. These responses were altered after MQEP intervention, the activation of the RAAS was inhibited, and the inhibition of type H blood vessel formation was restored.

### MQEP exerted an ameliorative effect on bone turnover regulatory factors and bone structure in an osteoporosis mouse model

**Figure 3:**
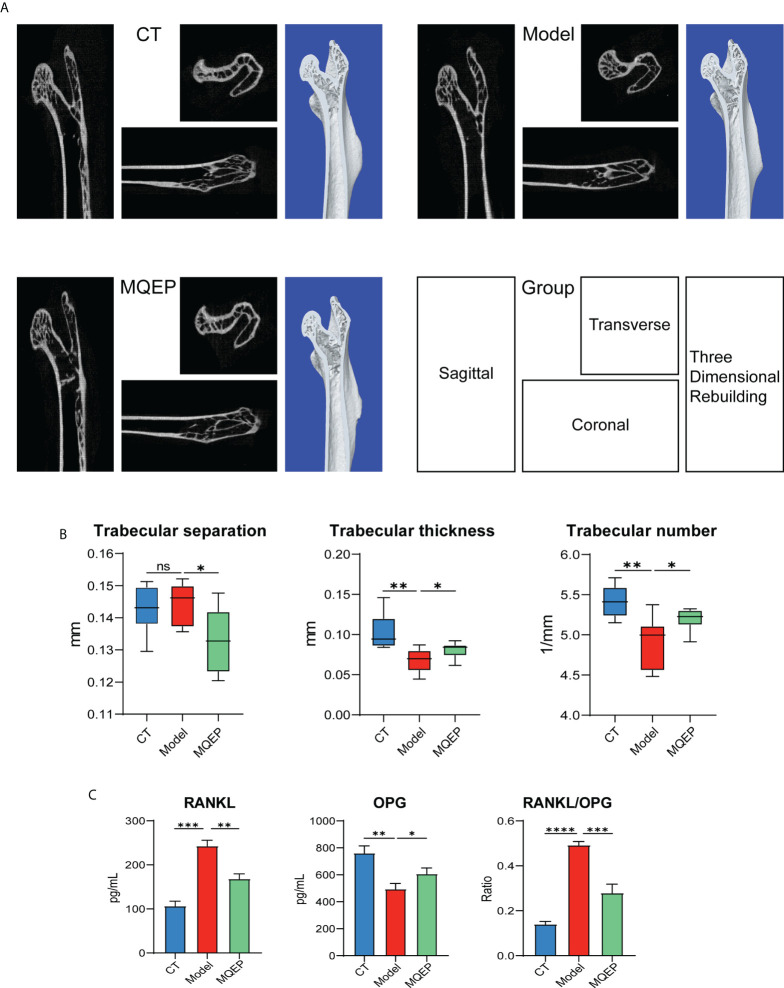
MQEP exerts an ameliorative effect on bone turnover regulatory factors and bone structure. **(A)** Micro-CT was used to evaluate the femur microstructure. The bone structure was observed from three perspectives and reconstructed in three dimensions. **(B)** Micro-structure measurements were calibrated to the manufacturer’s calcium hydroxyapatite phantom and analyzed using the manufacturer’s evaluation software. **(C)** Collection of serum and detection of relevant indicators using Elisa. ns, not significant, *p < 0.05, **p < 0.01, ***p < 0.001.

Micro-CT is one of the most intuitive and commonly used methods to assess basic skeletal conditions. We observed that after prolonged and continuous use of GCs, the microstructure of mouse femur in the model group was disrupted, and the bone trabeculae-related indexes (trabecular spacing, trabecular thickness, and trabecular number) used to evaluate bone strength indexes showed adverse progression **(** **Figures 3A, B** **)**. The spacing of the trabeculae was broadened, the thickness of the trabeculae was thinned, and the number of trabeculae was reduced, indicating that the model group mice had experienced bone loss and osteoporosis after prolonged and continuous use of GCs. In addition, we collected supernatant of femoral tissue from mice. We detected some indicators for evaluating the degree of local bone resorption and the expression of RANKL and OPG for evaluating the degree of local bone resorption **(** **Figure 3C** **)**. We observed that the expression of OPG decreased, RANKL increased, and the RANKL/OPG ratio increased after prolonged and continuous GC use. However, we observed a considerable change after the intervention with MQEP; the bone microstructure was restored, the number of trabeculae increased, the spacing of trabeculae became smaller, and the thickness of trabeculae increased, and the overall bone strength was improved considerably. Moreover, the upregulation of OPG expression, the downregulation of RANKL expression, and the decrease in the RANKL/OPG ratio suggested that after the intervention with MQEP, bone strength was partially restored, and the process of bone loss was prevented.

### The intervention of MQEP promoted the formation of type H blood vessels

**Figure 4:**
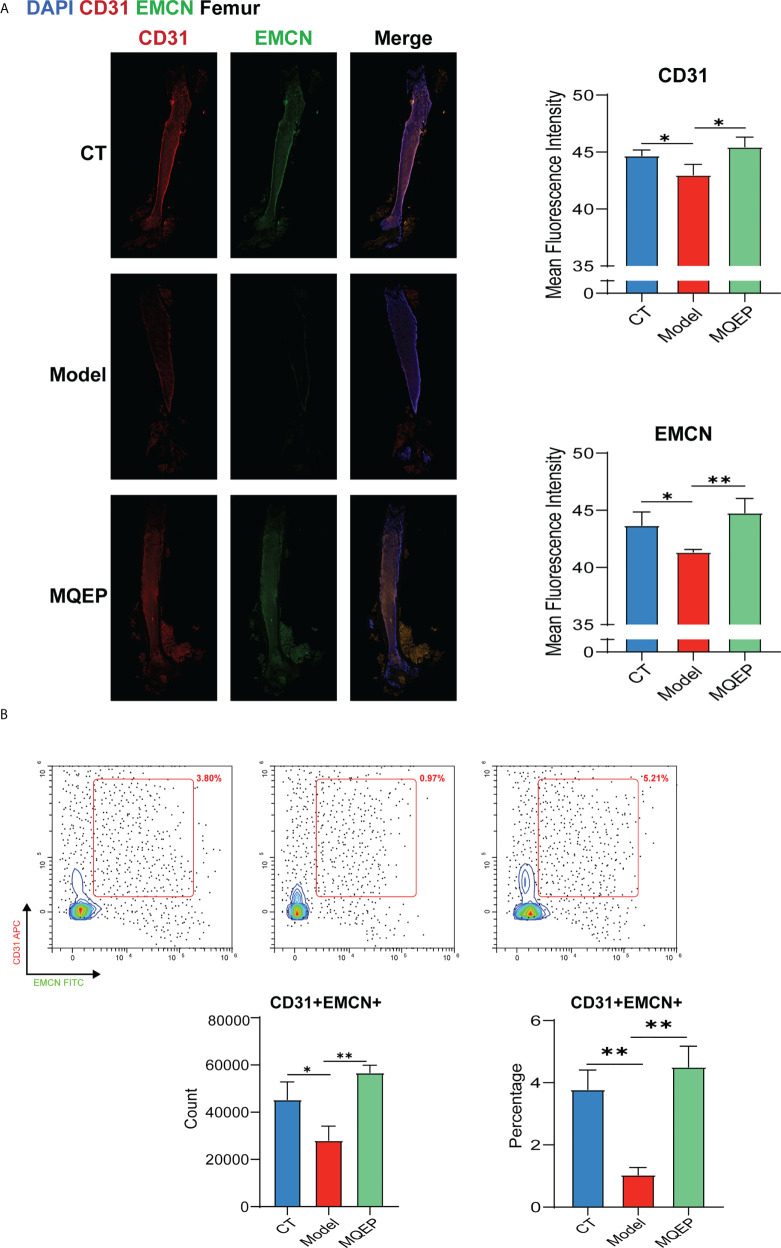
The intervention of MQEP promotes the formation of type H blood vessels. **(A)** Tissue immunofluorescence was performed to observe type H blood vessel formation, and the images were quantified and statistically analyzed. **(B)** Flow cytometry was performed to detect the specificity indexes of type H blood vessel formation. *p < 0.05, **p < 0.01.

Type H blood vessels are a specific vascular subtype shown to promote osteoblast activity and induce bone formation and are an important vehicle for building vascular-osteogenic couplings. To further confirm the ability of MQEP intervention to promote angiogenesis, especially in type H blood vessels, we labeled type H blood vessels using flow cytometry and immunohistochemistry. Immunohistochemistry showed that the expression of CD31^hi^EMCN^hi^ in the femurs of mice in the model group was inhibited after prolonged and continuous use of GCs **(** **Figure 4A** **)**, while flow cytometry showed that the percentage of CD31^hi^EMCN^hi^ cells in the femoral osteocytes of the model group of mice with prolonged continuous use of GCs was significantly decreased **(** **Figure 4B** **)**. After treatment with MQEP, the expression of CD31^hi^EMCN^hi^ in the femur increased as well as the percentage of this cell population. The immunohistochemistry results were consistent with the flow cytometry results. Finally, MQEP alleviated type H blood vessel injury caused by the prolonged and continuous use of GCs. This is the first study to demonstrate that MOEF promotes the proliferation of type H blood vessels.

## Discussion

Osteoporosis occurs due to a negative balance between bone formation and resorption due to multiple causes, with imbalances in bone reconstruction and bone loss, ultimately leading to reduced bone mass, damage to bone microarchitecture, and increased bone fragility. During this stage, patients usually do not experience any discomfort or only have non-specific symptoms and therefore do not undergo active intervention. The occurrence of osteoporosis is influenced by several factors, among which the long-term use of GC is one of the main risk factors (2). Some studies have confirmed that even small daily doses of GCs increase the risk of osteoporotic fractures and that the incidence of osteoporosis is higher in patients who receive long-term GC therapy (>1 year) (18). Previous studies on the mechanisms by which GC causes osteoporosis focused on the interference with endocrine metabolism and bone-associated cells. GC use leads to disruption of hormone metabolism in the body, resulting in poor bone mineralization and impaired bone formation, as well as decreased muscle strength and an increased risk of falls (19). GC mainly affects osteocytes, osteoblasts, and osteoclasts (20, 21). The effects of GC on osteocytes are mainly due to the release of sclerostin and dickkopf-1 by osteocytes and inhibition of the Wnt pathway to damage osteogenesis. GC also leads to increased autophagy and apoptosis in osteocytes and changes in bone microarchitecture. The effect on osteoclasts is reflected in the stimulation of RANKL and the inhibition of OPG. At the same time, GC increases the number and viability of osteoclasts by stimulating the production of colony-stimulating factors and promoting the differentiation of hematopoietic stem cells into osteoclast precursor cells; the effect on osteoblasts is the opposite. GC inhibits the production and release of transcription factors related to the Wnt pathway and inhibits osteoblast division by blocking the osteoblast cycle. In addition, GC disrupts the homeostasis of bone formation and resorption by inducing apoptosis in osteoblasts (22–24). GC also affects blood vessels as it inhibits bone angiogenesis and reduces the blood supply in the microtubular system of the bone lacunae. Moreover, patients with long-term GC use are of advanced age, have underlying or autoimmune diseases and other confounding factors, and have poor skeletal and vascular conditions. Fractures may recur after surgery. Therefore, improving vascular conditions and promoting angiogenesis is another effective strategy to promote bone formation and inhibit bone resorption (25, 26).

Blood vessel formation and vascular condition are influenced by several factors. The RAAS activation damage and oxidative stress damage are two major factors that inhibit blood vessel formation and cause deterioration of the vascular condition. Closely related to bone metabolism is the activation of RANKL/RANK/OPG signaling. The RANKL/RANK/OPG signaling pathway is closely related to bone metabolism and can affect the formation of blood vessels. GC use leads to activation of the RAAS system, which inhibits vascular endothelial cell regeneration and induces apoptosis. Endothelial cell injury leads to oxidative stress, and the release of reactive oxygen species further aggravates a vascular injury, creating a vicious cycle. Blockade of RANKL effectively reduces vascular endothelial cell injury and oxidative stress (27, 28). Moreover, it has been experimentally demonstrated that RANKL inhibits microvascular endothelial growth factor-induced angiogenesis and OPG increases the rate of microvascular endothelial cell proliferation; thus, OPG is a positive regulator of microangiogenesis, and RANKL is an inhibitor of angiogenesis (29). The mutual synergy between these two signaling pathways exacerbates skeletal vascular injury. The femur is characterized by its specific structural location of stress concentration, resulting in increased intravascular pressure and a specific type of mid-terminal arterial blood supply with low collateral circulation. This makes the femur more susceptible to inadequate blood supply or vascular damage than other bone tissues during long-term GC use. Therefore, improving blood supply or promoting localized angiogenesis in the femur during bone maintenance is important. Good vascular condition and the generation of new blood vessels are important for bone formation and fracture healing.

Type H blood vessels are a specific type of blood vessel identified recently as being highly correlated formation (30). They were negatively correlated with age; the number of type H blood vessels decreased significantly with age (10). In addition, type H blood vessels were negatively correlated with estrogen levels (31); the number of type H blood vessels decreased significantly in an estrogen-deficient mouse model (32). High expression of runt-related transcription factor 2 and osterix around type H blood vessels, two transcription factors that are key for bone formation, have also been observed. Moreover, the extracellular matrix of type H blood vessels is capable of secreting various molecules that stimulate osteoblast differentiation and proliferation through the Notch signaling pathway. The two major signaling pathways involved in regulating type H blood vessel formation are the HIF-1a and VEGF signaling pathways. These two signaling pathways have positive interactions with each other (33). Local vascular injury leads to hypoxia, which stimulates HIF-1a expression, and high expression of HIF-1a protein promotes type H blood vessel formation (34). HIF-1a increase also directly induces the transcription and translation of VEGF. VEGF is a major regulator of angiogenesis, and its ability to promote angiogenesis by facilitating the migration of the extravascular matrix has been extensively studied and confirmed (35). Changes in type H blood vessels have become an early marker for assessing skeletal status, and inducing type H blood vessel formation is emerging as a potential target for improving bone quality.

MQEP is a traditional medicine with multiple uses in China. It is currently used mainly for back pain caused by osteoporosis and is included in the Chinese osteoporosis treatment guidelines. It has various pharmacological activities, such as anti-inflammatory, anti-aging, and estrogenic properties (11, 36). Although MQEP has been used in China for more than 1,300 years, its mechanism of action is still unclear and is only explained based on TCM theories; its clinical use relies more on the experience of TCM doctors. With the advancement of modern research in TCM, modern pharmacological studies on the regulation of bone metabolism by MQEP have also emerged (37). Current research suggests that MQEP can improve the femoral microstructure and promote femoral vascular microcirculation in patients with non-traumatic osteonecrosis of the femoral head by regulating adiponectin levels, bone morphogenetic protein 2, OPG, and other key transcription factors affecting bone metabolism (38). MQEP can also exert anti-aging effects by enhancing overall antioxidant capacity and improving hepatic and renal serological indicators (39).

In this study, we observed that MQEP promotes angiogenesis by inhibiting the activation of the RAAS and RANKL/RANK/OPG systems. By inhibiting these two pathways, MQEP alleviates microangiogenesis and circulatory disorders and promotes angiogenesis. One specific mechanism may be that MQEP inhibits the expression of AT1R/AT2R/ACE to suppress the activation of the RAAS caused by long-term GC use. In addition, MQEP inhibited the activation of the RANKL/RANK/OPG signaling pathway, reduced RANKL expression, and reversed the decrease in OPG expression caused by long-term GC use, thus reducing the ratio of RANKL/OPG and causing the negative balance of bone resorption over bone formation, bone loss, and osteoporosis to be recovered to some extent. Downregulation of RANKL expression and upregulation of OPG expression could promote osteoblast proliferation and viability and inhibit osteoclast proliferation and viability. Microvascular dysfunction is ameliorated by the inhibition of the RAAS and RANKL/RANK/OPG systems, and the simultaneous restoration of vascular conditions and angiogenesis promote local bone reconstruction. The other mechanism may be to enhance the activity of vascular endothelial cells and promote the proliferation of type H blood vessels by upregulating the expression of HIF-1a and VEGF, thus enhancing vascular osteogenic coupling. The combined effects of the two mechanisms and three pathways alleviate bone loss and osteoporosis caused by long-term GC use, improve the bone microstructure and the overall skeletal condition by increasing bone mineral density and improving the number, density, and thickness of bone trabeculae, fully reflecting the multiple pathways and targets of MQEP. Although MQEP has multiple components and targets and exerts its pharmacological effects through different mechanisms, its effect on improving microvascular conditions and promoting type H blood vessel formation is apparent, and the outcome is positive. In our previous study, we performed a targeted metabolomic analysis of the components of the aqueous decoction of MQEP to obtain small molecules. Although we obtained information on the small molecule compounds in MQEP by mass spectrometry and also examined the regulatory mechanism of *Isopsoralen* at the transcriptional level, we did not validate it at the protein level, this is a regret of the present study. Therefore, our future research will be to select small molecules with good bioavailability, and obvious biological effects from targeted metabolomics. This is one of our ongoing research projects, and we look forward to even more encouraging results in the future.

**Figure 5:**
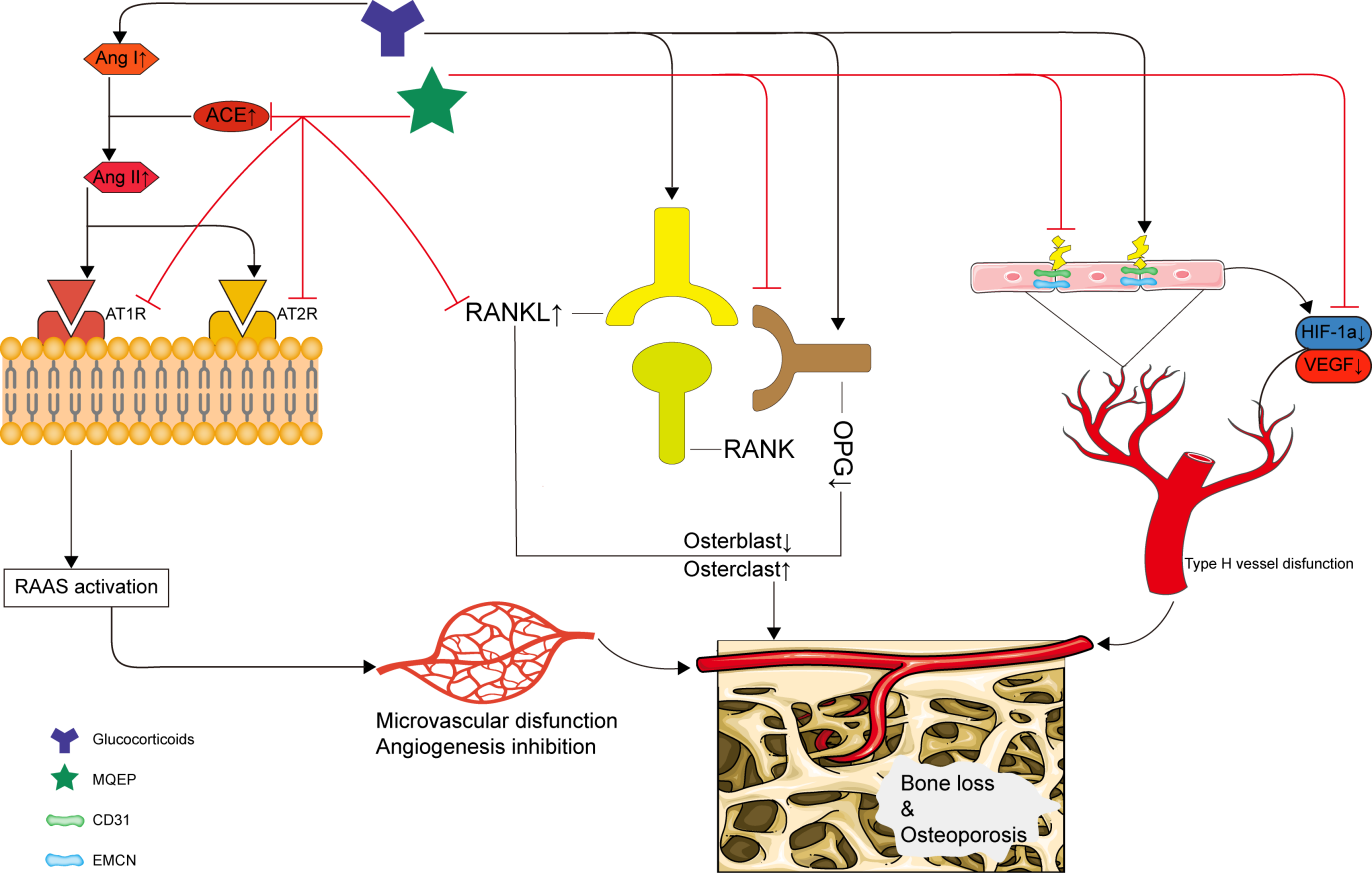
Schematic depiction of the mechanism by which the MQEP exerts its anti-osteoporosis and bone loss prevention effects by enhancing vessel formation.

In summary, our study clarified a novel pharmacological mechanism by which MQEP exerts anti-osteoporosis effects and prevents bone loss in clinical settings. We demonstrated for the first time that MQEP could inhibit the activation of the RAAS and RANKL/RANK/OPG systems and promote type H blood vessel formation, effectively preventing bone loss and osteoporosis caused by long-term GC use. Our findings provide strong evidence supporting the clinical use of MQEP (**Figure 5**). We also further explored the specific mechanism of *Isopsoralen*, one of the active components of MQEP.

## Data availability statement

The original contributions presented in the study are included in the article/**Supplementary Material**. Further inquiries can be directed to the corresponding author.

## Ethics statement

The animal study was reviewed and approved by Institutional Animal Care and Use Committee of Tongji Medical College, Huazhong University of Science and Technology.

## Author contributions

BS, JL, CM, XX and DH conceived the idea for the study and provided critical revision of the manuscript. CM, XX, JR, JZ and DH collected the information. BS, JL, CM, XX and DH participated in study design, supervising, writing and drafting of the manuscript. All authors read and approved the final manuscript.

## Funding

This study was partially funded by the National Natural Science Foundation of China (project numbers: 81974546, 82174182, 81974249, 82004201, and 82004098).

## Acknowledgments

The authors would like to thank Editage (www.editage.cn) for English language editing.

## Conflict of interest

The authors declare that the research was conducted without any commercial or financial relationships that could be construed as a potential conflict of interest.

## Publisher’s note

All claims expressed in this article are solely those of the authors and do not necessarily represent those of their affiliated organizations, or those of the publisher, the editors and the reviewers. Any product that may be evaluated in this article, or claim that may be made by its manufacturer, is not guaranteed or endorsed by the publisher.

## Abbreviations

MQEP, modified Qing’ e Pills; GC, Glucocorticoid; RAAS, renin-angiotensin-aldosterone system; RANK(L), nuclear factor-kappa B (ligand); OPG, osteoprotegerin; EMCN, endomucin; HIF-1a, hypoxia-inducible factor 1 alpha; VEGF, vascular endothelial growth factor; DEX, dexamethasone; HUVEC, human umbilical vein endothelial cells; AT1(R), angiotensin II type 1 (receptor); AT2(R), angiotensin II type 2 (receptor); ACE, angiotensin converting enzyme; CT, computed tomography; DPBS, Dulbecco’s phosphate-buffered saline.
