# Supplementary figures and images for: Modified Qing’ e Pills exerts anti-osteoporosis effects and prevents bone loss by enhancing type H blood vessel formation
Source: Front Endocrinol (Lausanne). 2022 Sep 6;13:998971. doi: 10.3389/fendo.2022.998971 (PMC9485463; doi:10.3389/fendo.2022.998971)

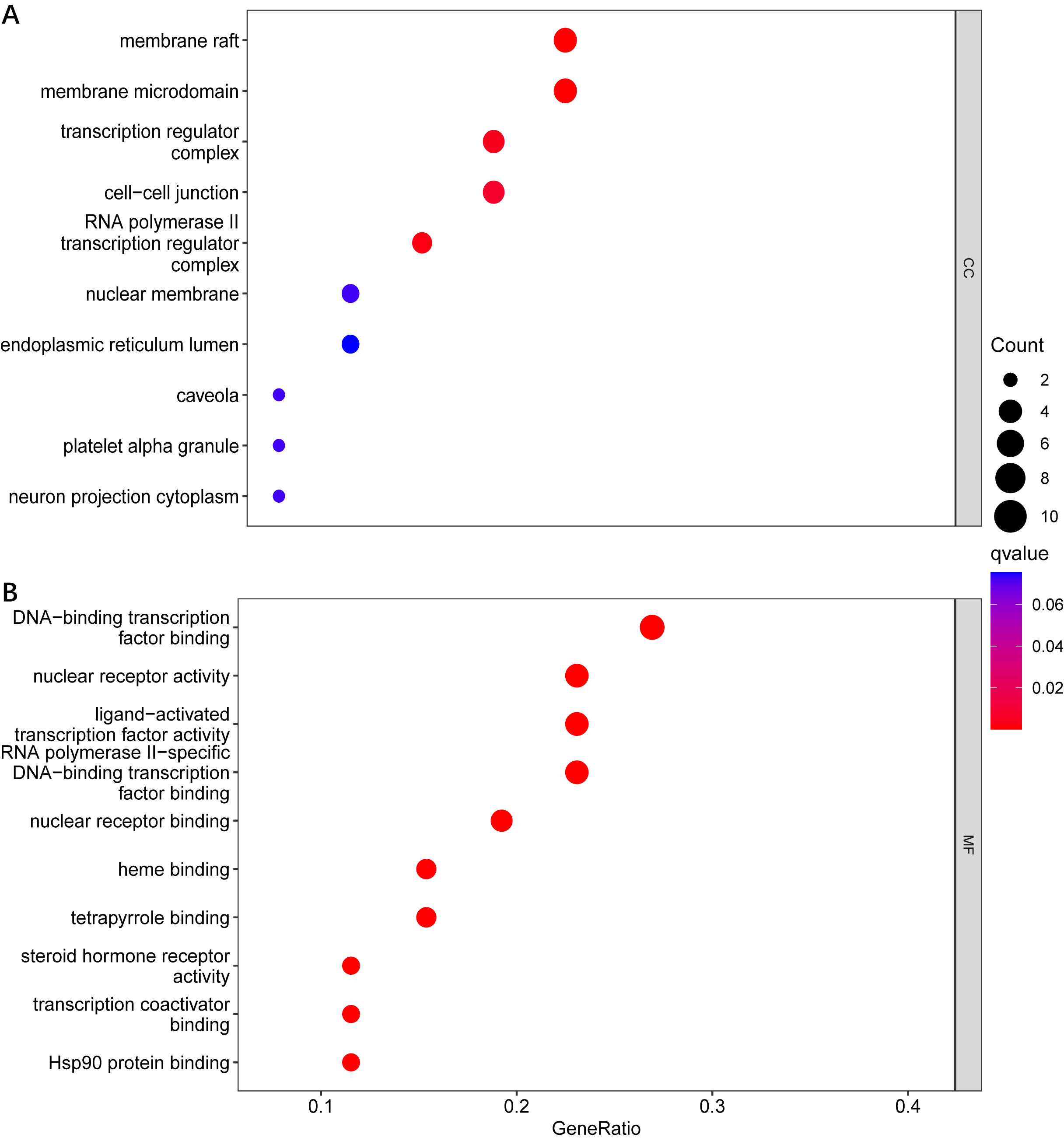

Supplement: Supplementary file 1 [file Image_1.tif]
